# Diagnostic Value of a Wearable Continuous Electrocardiogram Monitoring Device (AT-Patch) for New-Onset Atrial Fibrillation in High-Risk Patients: Prospective Cohort Study

**DOI:** 10.2196/45760

**Published:** 2023-09-18

**Authors:** Ju-Seung Kwun, Jang Hoon Lee, Bo Eun Park, Jong Sung Park, Hyeon Jeong Kim, Sun-Hwa Kim, Ki-Hyun Jeon, Hyoung-won Cho, Si-Hyuck Kang, Wonjae Lee, Tae-Jin Youn, In-Ho Chae, Chang-Hwan Yoon

**Affiliations:** 1 Cardiovascular Center Seoul National University Bundang Hospital Seongnam-si Republic of Korea; 2 Department of Internal Medicine Kyungpook National University Hospital Daegu Republic of Korea; 3 Department of Internal Medicine Sihwa Medical Center Siheung-si Republic of Korea

**Keywords:** arrhythmias, atrial fibrillation, wearable electronic device, patch electrocardiogram monitor, electrocardiogram, adult, AT-Patch, heart failure, mobile phone

## Abstract

**Background:**

While conventional electrocardiogram monitoring devices are useful for detecting atrial fibrillation, they have considerable drawbacks, including a short monitoring duration and invasive device implantation. The use of patch-type devices circumvents these drawbacks and has shown comparable diagnostic capability for the early detection of atrial fibrillation.

**Objective:**

We aimed to determine whether a patch-type device (AT-Patch) applied to patients with a high risk of new-onset atrial fibrillation defined by the congestive heart failure, hypertension, age ≥75 years, diabetes mellitus, stroke, vascular disease, age 65-74 years, sex scale (CHA_2_DS_2_-VASc) score had increased detection rates.

**Methods:**

In this nonrandomized multicenter prospective cohort study, we enrolled 320 adults aged ≥19 years who had never experienced atrial fibrillation and whose CHA_2_DS_2_-VASc score was ≥2. The AT-Patch was attached to each individual for 11 days, and the data were analyzed for arrhythmic events by 2 independent cardiologists.

**Results:**

Atrial fibrillation was detected by the AT-Patch in 3.4% (11/320) of patients, as diagnosed by both cardiologists. Interestingly, when participants with or without atrial fibrillation were compared, a previous history of heart failure was significantly more common in the atrial fibrillation group (n=4/11, 36.4% vs n=16/309, 5.2%, respectively; *P*=.003). When a CHA_2_DS_2_-VASc score ≥4 was combined with previous heart failure, the detection rate was significantly increased to 24.4%. Comparison of the recorded electrocardiogram data revealed that supraventricular and ventricular ectopic rhythms were significantly more frequent in the new-onset atrial fibrillation group compared with nonatrial fibrillation group (3.4% vs 0.4%; *P=*.001 and 5.2% vs 1.2%; *P<*.001), respectively.

**Conclusions:**

This study detected a moderate number of new-onset atrial fibrillations in high-risk patients using the AT-Patch device. Further studies will aim to investigate the value of early detection of atrial fibrillation, particularly in patients with heart failure as a means of reducing adverse clinical outcomes of atrial fibrillation.

**Trial Registration:**

ClinicalTrials.gov NCT04857268; https://classic.clinicaltrials.gov/ct2/show/NCT04857268

## Introduction

Stroke prevention, which is frequently associated with atrial fibrillation, is currently a leading global health concern [1]. Importantly, atrial fibrillation increases the risk of stroke 5-fold and accounts for approximately 25% of cryptogenic strokes [2]. These events are largely preventable through the use of anticoagulant therapy, and the advent of nonvitamin K antagonist oral anticoagulants has resulted in better patient adherence and compliance in addition to better outcomes [3,4]. Despite early detection and accurate diagnosis of arrhythmias being crucial for preventing adverse outcomes [5], atrial fibrillation is often both asymptomatic and intermittent, making it difficult to capture these episodic events [6,7].

While conventional electrocardiogram (ECG) monitoring devices, including multilead portable ECG monitoring devices, event-detection monitoring devices, and implantable ECG monitoring devices, are useful for the early detection of atrial fibrillation, they also have considerable drawbacks including the requirement for multiple outpatient visits as well as invasive device implantation [8]. To overcome these disadvantages, several newer-generation ECG monitoring devices with advanced technologies have been developed [9]. Of these, Zio Patch (iRhythm Technologies) is a single-use patch-type ECG monitoring device that is capable of continuously monitoring a patient’s ECG signal for 2 weeks, as demonstrated through its application to more than 400,000 patients [10]. In comparison, AliveCor KardiaMobile (AliveCor Inc) is a smartphone-connected ECG monitoring device that can measure the single-lead ECG signal of a patient and following Food and Drug Administration clearance in 2014, has been widely used to detect symptomatic arrhythmias such as atrial fibrillation [11]. Unlike conventional ECG monitoring devices, these new-generation ECG monitoring devices allow for increased monitoring duration, wireless data transfer, and their portable size substantially decreases the impact on the daily life of patients [12].

In addition to these devices, the AT-Patch (ATsens) is a single-lead ECG monitoring device that is capable of continuously monitoring an ECG signal for up to 14 days and also enables the correlation of ECG changes and patient symptoms via a smartphone connection. Moreover, it has recently been demonstrated to have comparable diagnostic capability and safety to conventional ECG monitoring systems [13]. Despite this, the device is currently not widely used in real-world settings as it remains uncertain as to whether systematic screening for atrial fibrillation with cost- and labor-efficient patch devices, compared to traditional care, identifies older adults with previously undiagnosed atrial fibrillations more effectively or better improves their health outcomes.

Furthermore, several studies have indicated high performance rates when using the validated congestive heart failure, hypertension, age ≥75 years, diabetes mellitus, stroke, vascular disease, age 65-74 years, sex scale (CHA_2_DS_2_-VASc) score to predict new-onset atrial fibrillation, with over 10% of patients with CHA_2_DS_2_-VASc scores ≥2 having new-onset atrial fibrillations [14]. This study therefore sought to evaluate the validity of the CHA_2_DS_2_-VASc score for the early detection of new-onset atrial fibrillation in high-risk patients using the AT-Patch.

## Methods

### Ethics Approval

This nonrandomized, noncontrol, multicenter, and prospective cohort study was carried out between November 2020 and April 2022 and was reviewed and approved by the institutional review board of the Seoul National University Bundang Hospital (B-2009-634-003). The protocol was registered with the Clinical Research Information Service of the Korea Centers for Disease Control and Prevention, Ministry of Health and Welfare, Republic of Korea (KCT0005650) on December 2, 2020. It was registered at ClinicalTrials.gov (NCT04857268) for international access to the protocol on April 23, 2021.

### Eligibility Criteria

We enrolled 320 adults aged ≥19 years who visited 2 tertiary hospitals in Korea. Inclusion criteria were as follows: (1) patients who provided written and informed consent to participate and (2) those with CHA_2_DS_2_-VASc scores ≥2. The CHA_2_DS_2_-VASc score was calculated as follows: congestive heart failure, hypertension, diabetes mellitus, aged between 65 and 75 years, vascular disease, and female sex (1 point for each parameter), and stroke or transient ischemic attack and age >75 years (2 points for each). The maximum CHA_2_DS_2_-VASc score was 9 points. Congestive heart failure was defined as a previous diagnosis of heart failure (signs or symptoms of heart failure or objective evidence of reduced left ventricular ejection fraction ≤40%). Hypertension was defined as a systolic blood pressure greater than 140 mm Hg, diastolic blood pressure greater than 90 mm Hg, or prior antihypertensive drug use. Diabetes mellitus was defined as fasting glucose levels greater than 126 mg/dL or the presence of previous antidiabetic drug treatment. Vascular disease was defined as the prior occurrence of myocardial infarction, peripheral vascular disease, or aortic plaque [15]. The exclusion criteria were as follows: (1) previous diagnosis of atrial fibrillation; (2) implanted pacemaker, cardioverter-defibrillator, or any electrical device; (3) skin problems such as allergic contact dermatitis; and (4) female patients who were pregnant or lactating.

### AT-Patch Device

The AT-Patch is a single-use device that can continuously record the electrical activity of the heart. This single-lead, noninvasive ECG recorder is indicated for ambulatory ECG monitoring for up to 14 days (11 days if the device is connected to a smartphone via Bluetooth). It weighs only 13 g, has a size of 95.0 × 50.6 × 8.3 mm, and allows for uninterrupted recording during sleep and light physical activity, thereby offering user comfort and increased patient adherence.

A study coordinator placed this device on the patient’s left pectoral region, tilted 45° inward. To prevent noise or signal loss, the skin was cleansed using a 70% ethanol solution prior to attachment. Participants were instructed to wear the adhesive patch for as long as possible in order to obtain ECG data for up to 11 days. A continuous ECG signal was recorded for 11 days and stored on a memory card. Following this, the device was linked to a computer, and the data were subsequently downloaded and analyzed using AT-report, a specific program provided by ATsens Co, Ltd.

### Trial Schedule

Clinical assessments were scheduled for baseline and days 11, 90, and 180. During the baseline assessment, we obtained the participants’ demographic data, past and present medical and drug administration history, and conducted physical examinations (height, body weight, vital signs such as systolic and diastolic blood pressure, and pulse), laboratory parameters, and 12-lead ECG results (if test results within the prior 4 weeks were available, they were used instead). For eligible participants, we attached the experimental AT-Patch device according to the institutional Good Clinical Practice for medical devices and recorded the date and time. During the following visit (day 11±5), we detached the device from the patient and analyzed the recorded data for atrial fibrillation detection. During the third visit (day 90±15), routine physical examinations (blood pressure and pulse) and 12-lead ECG were performed. Finally, during the last visit (day 180±15), the participants repeated the clinical assessments performed during the third visit. If atrial fibrillation or other types of arrhythmias were detected via either the AT-Patch or 12-lead ECG, the investigator scheduled the individual for further assessment.

### End Points

The primary end point was the incidence of newly diagnosed atrial fibrillation as defined by ≥30 seconds of atrial fibrillation or flutter detected by the device. ECG data were reviewed by 2 independent cardiologists, and the diagnosis was confirmed when both reviewers were in agreement. The secondary end points were (1) a new clinical diagnosis of atrial fibrillation by 12-lead ECG at a scheduled or unscheduled follow-up visit and (2) death, acute myocardial infarction, stroke, or systemic embolic events during the 6-month monitoring period.

### Sample Size Calculation

Previous studies reported the incidence of new-onset atrial fibrillation as 0.76 per 100 person-years in adults aged ≥50 years which gradually increased to 0.17-6.71, according to the CHA_2_DS_2_-VASc score [14] We hypothesized that the AT-Patch device would be capable of detecting 8% new-onset atrial fibrillation in high-risk patients with a CHA_2_DS_2_-VASc score ≥2. This is more than 8 times the expected incidence rate in these patients. We set the type I error to 0.05 and the confidence limit to 3% which resulted in 315 participants. Finally, we set the attrition rate to 1.5% which resulted in the final sample size of 320 participants.

### Statistical Analyses

Data are presented as numbers and frequencies for categorical variables and as means and SDs for continuous variables. The incidence of newly diagnosed atrial fibrillation is described as the proportion and 95% CI. A 2-sided *P* value of <.05 was considered statistically significant. The Cohen κ coefficient was applied to evaluate interobserver reliability between the 2 independent cardiologists’ interpretations. McNemar’s test was applied to a 2 × 2 contingency table to determine the detection power of the AT-Patch device in comparison with the 12-lead ECG. For comparisons between patients with or without atrial fibrillation, the chi-square test (or Fisher’s exact test when any expected count was <5 for a 2 × 2 or 2 × 4 table) was performed for categorical variables, and a 2-sample *t* test or the Wilcoxon rank sum test was applied for continuous variables, dependent upon whether the data followed a normal distribution. Statistical analyses were performed using R software (version 3.1.0; R Foundation for Statistical Computing).

## Results

### Cardiac Arrhythmia Detection Rates

A total of 320 participants were enrolled in this study. The incidence of arrhythmias, as detected by the AT-Patch and subsequently confirmed by 2 cardiologists (C1 and C2), is shown in Table 1.

**Table 1:** Incidence of arrhythmias detected using AT-Patch and confirmed by independent cardiologists^a^.

	C1^b^, n (%)	C2^c^, n (%)	C1 and C2, n (%)
Supraventricular tachycardia	186 (58.1)	182 (56.9)	149 (46.6)
Nonsustained ventricular tachycardia	24 (7.5)	16 (5)	13 (4.1)
Ventricular tachycardia	0 (0)	0 (0)	0 (0)
Ventricular fibrillation	0 (0)	0 (0)	0 (0)
Atrial fibrillation	14 (4.4)	12 (3.8)	11 (3.4)

^a^Cohen κ: 0.840 (0.79-0.89); *P*<.001.

^b^C1: cardiologist 1.

^c^C2: cardiologist 2.

**Figure 1:**
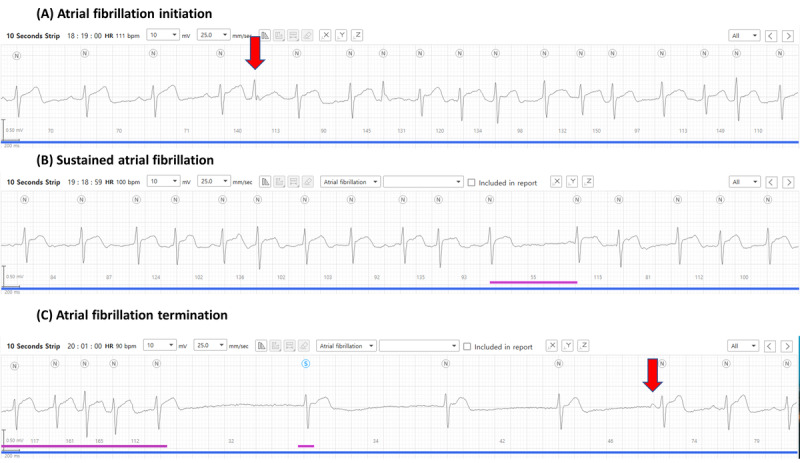
Examples of (A) initiation of atrial fibrillation (arrow: a preceding premature beat), (B) sustainment, and (C) termination (arrow: appearance of the first P wave) as detected by the AT-Patch.

**Figure 2:**
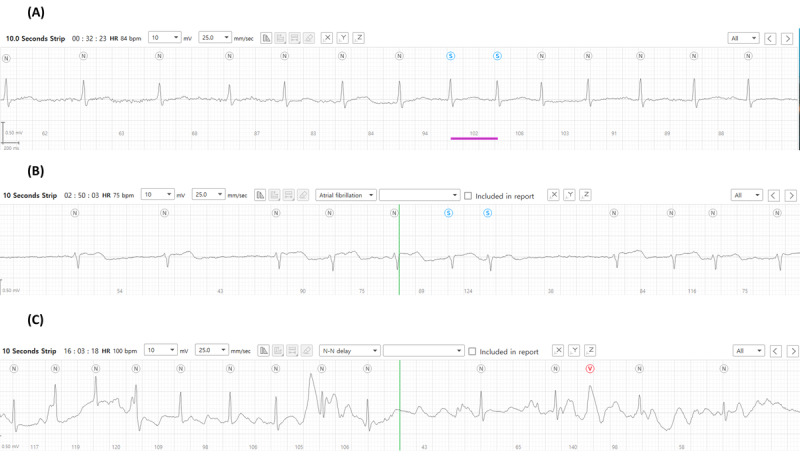
Three representative examples of arrhythmic events that were differentially diagnosed as atrial fibrillation or atrial tachycardia by 2 cardiologists.

It was determined that atrial fibrillation occurred in 4.4% (14/320) and 3.8% (12/320) of patients by cardiologist 1 (C1) and cardiologist 2 (C2), respectively, with example recordings of a detected episode shown in Figure 1. It is evident that this episode of atrial fibrillation began following a premature beat (Figure 1A) and thereafter presented as irregular heartbeats in the absence of P waves (Figure 1B). Moreover, the termination of atrial fibrillation as indicated by prolonged sinus pauses and sinus rhythm with clear P waves was captured by the patch device (Figure 1C). In total, 11 (3.4%) patients were concordantly diagnosed with atrial fibrillation by both cardiologists, while 3 arrhythmic events were discordantly diagnosed. In total, 1 cardiologist interpreted them as atrial fibrillations as they showed short episodes of irregular and uncertain P waves (Figures 2A-2C). However, the other cardiologist determined these episodes to be atrial tachycardia. Despite this, the overall Cohen κ coefficient was 0.840, which confirmed the almost perfect reliability of the 2 cardiologists’ interpretations. Detailed information on atrial fibrillation episodes detected by AT-Patch is presented in Table S1 in Multimedia Appendix 1. Furthermore, 3 participants, in whom atrial fibrillation had not been detected by the patch device, were diagnosed with new-onset atrial fibrillation by the 12-lead conventional ECG at 3- or 6-month follow-up visits (Table 2).

**Table 2:** Detection of new-onset atrial fibrillation by AT-Patch versus conventional 12-lead ECG^a^.

		AT-Patch				
		(+)^b^		(–)^b^		Total
**Conventional 12-lead ECG**						
	(+)	0	3 (0.94%, 95% CI 0.24-2.95)		3	
	(–)	11 (3.44%, 95% CI 1.82-6.24)	306		317	
	Total	11	309		320	

^a^ECG: electrocardiogram.

^b^The 95% CI for the proportion of patients with AT-Patch (+) and (–) are shown in parentheses.

**Figure 3:**
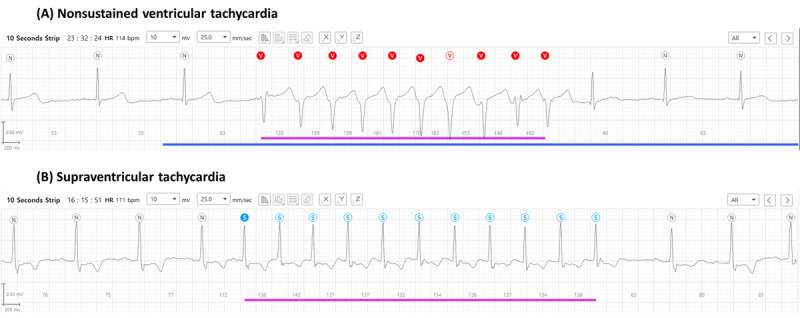
Examples of detected arrhythmias. (A) Nonsustained ventricular tachycardia. (B) Supraventricular tachycardia.

Overall, the total number of patients with new-onset atrial fibrillation was 4.4% (14/320). Interestingly, none of the participants presented with atrial fibrillation with both the patch monitor and 12-lead ECG. Moreover, the cardiologists determined that nonsustained ventricular tachycardia occurred occasionally, in 4.1% (13/320) of patients, and supraventricular tachycardia occurred more frequently, in 46.6% (149/320) of patients (Figure 3). No sustained ventricular tachycardia or fibrillation was detected using the patch device.

### Characteristics of the Participants Newly Diagnosed With Atrial Fibrillation

Across the 320 participants with a CHA_2_DS_2_-VASc score ≥2, the mean age was 73.3 (SD 7.8) years, and the average CHA_2_DS_2_-VASc score was 3.6 (SD 1.2; Table 3). This study’s population consisted of 73.8% (236/320) of patients with hypertension, 40% (128/320) with diabetes mellitus, 6.2% (20/320) with previous heart failure, and 11.9% (38/320) with a history of stroke. In addition, 51.9% (166/320) of patients received β-blockers, and 65.9% (211/320) of patients received renin-angiotensin system inhibitors. Patients with new-onset atrial fibrillation detected by the AT-Patch alone had a history of heart failure significantly more often than those without (n=4/11, 36.4% vs n=16/309, 5.2%, respectively; *P*=.003). Interestingly, among the administered medications, the use of angiotensin-converting enzyme inhibitors was significantly different between patients with and without episodes of atrial fibrillation as detected by the AT-Patch device alone (n=4/11, 36.4% vs n=36/309, 11.7%, respectively; *P*=.04). When we evaluated patients with new-onset atrial fibrillation detected by AT-Patch or 12-lead ECG, those with previous heart failure were significantly different (n=6/14, 42.9% vs n=14/306, 4.6%; *P*<.001).

**Table 3:** Profile of this study’s population and comparison between the atrial fibrillation group and the nonatrial fibrillation group.

Characteristics		Total participants		Atrial fibrillation by AT-Patch							Atrial fibrillation by AT-Patch or 12-lead ECG^a^				
				Yes (N=11)		No (N=309)		*P* value		Yes (N=14)			No (N=306)		*P* value
Age (years), mean (SD)		73.3 (7.83)		71.9 (11.41)		73.3 (7.70)		.96		73.7 (11.06)			73.24 (7.68)		.46
**Age groups, n (%)**															
	<55	N/A^b^	1 (9.1)		9 (2.9)		N/A		1 (7.1)			9 (2.9)		N/A	
	55-64	N/A	2 (18.2)		15 (4.9)		N/A		2 (14.3)			15 (4.9)		N/A	
	65-74	N/A	2 (18.2)		139 (45)		N/A		3 (21.4)			138 (45.1)		N/A	
	>75	N/A	6 (54.5)		146 (47.2)		N/A		8 (57.1)			144 (47.1)		N/A	
Sex (male), n (%)		181 (56.6)		5 (45.5)		176 (57)		.54		8 (57.1)			27 (87.1)		>.99
BMI (kg/m^2^), mean (SD)		24.8 (3.21)		26.2 (4.15)		24.8 (3.17)		.13		25.8 (4.06)			24.8 (3.17)		.73
Heart rate (beats/min), mean (SD)		67.8 (8.60)		68.1 (10.38)		67.7 (8.55)		.98		67.4 (9.46)			67.8 (8.58)		.77
Hypertension, n (%)		236 (73.8)		8 (72.7)		228 (73.8)		>.99		10 (71.4)			226 (73.9)		.77
Diabetes mellitus, n (%)		128 (40)		5 (45.5)		123 (39.8)		.76		5 (35.7)			123 (40.2)		.96
Dyslipidemia, n (%)		134 (41.9)		5 (45.5)		129 (41.7)		>.99		6 (42.9)			128 (41.8)		>.99
Heart failure, n (%)		20 (6.2)		4 (36.4)		16 (5.2)		.003		6 (42.9)			14 (4.6)		<.001
Previous MI^c^, n (%)		72 (22.5)		1 (9.1)		71 (23)		.47		2 (14.3)			70 (22.9)		.74
**Smoking**, n (%)								>.99							.74
	Current smoker	38 (11.9)	1 (9.1)		37 (12)		N/A		2 (14.3)			36 (11.8)		N/A	
	Former smoker	102 (31.9)	3 (27.3)		99 (32)		N/A		5 (35.7)			97 (31.7)		N/A	
	Never smoker	180 (56.2)	7 (63.6)		173 (56)		N/A		7 (50)			173 (56.5)		N/A	
History of stroke, n (%)		38 (11.9)		1 (9.1)		37 (12)		>.99		1 (7.1)			37 (12.1)		>.99
CKD^d^, n (%)		24 (7.5)		2 (18.2)		22 (7.1)		.20		3 (21.4)			21 (6.9)		.08
**Medications**, n (%)															
	Aspirin	183 (57.2)	4 (36.4)		179 (57.9)		.22		5 (35.7)			178 (58.2)		.17	
	P2Y12 inhibitors	119 (37.2)	6 (54.5)		117 (38.1)		.34		8 (57.1)			111 (36.3)		.20	
	ACE inhibitors^e^	40 (12.5)	4 (36.4)		36 (11.7)		.04		4 (28.6)			36 (11.8)		.08	
	ARB^f^	171 (53.4)	4 (36.4)		167 (54)		.40		6 (42.9)			165 (53.9)		.59	
	β-blockers	166 (51.9)	6 (54.5)		160 (51.8)		>.99		7 (50)			159 (52)		>.99	
	Calcium channel blockers	130 (40.6)	3 (27.3)		126 (41)		.55		4 (28.6)			126 (41.2)		.51	
	Statins	262 (81.9)	9 (81.8)		253 (81.9)		>.99		11 (78.6)			251 (82)		.73	
CHA_2_DS_2_-VASc^g^ score, mean (SD)		3.6 (1.25)		3.8 (1.72)		3.5 (1.23)		.65		3.8 (1.53)			3.5 (1.23)		.55

^a^ECG: electrocardiogram.

^b^N/A: not applicable.

^c^MI: myocardial infarction.

^d^CKD: chronic kidney disease.

^e^ACE inhibitors: angiotensin-converting enzyme inhibitors.

^f^ARB: angiotensin receptor blocker.

^g^CHA_2_DS_2_-VASc: congestive heart failure, hypertension, age ≥75 years, diabetes mellitus, stroke, vascular disease, aged 65-74 years, sex scale.

We determined that a CHA_2_DS_2_-VASc score ≥4 increased the detection rate of atrial fibrillation (Table 4), but not significantly (4/167, 2.4% vs 7/153, 4.6%; *P*=.29). However, when we combined a CHA_2_DS_2_-VASc score ≥4 and previous heart failure, the detection rate significantly increased to 24.4% (*P*=.003).

**Table 4:** Important predictive features for atrial fibrillation.

			Heart failure			
			No, n/N (%)	Yes, n/N (%)		Sum, n/N (%)
**CHA_2_DS_2_-VASc^a^**						
	<4	4/164 (2.4)		0/3 (0)	4/167 (2.4)	
	≥4	3/136 (2.4)		4/17 (24.4)	7/153 (4.6)	
	Sum	7/300 (2.3)		4/20 (20)	11/320 (3.4)	

^a^CHA_2_DS_2_-VASc: congestive heart failure, hypertension, age ≥75 years, diabetes mellitus, stroke, vascular disease, age 65-74 years, sex scale.

### Device Wear Time and Analyzable Data

The average recording time for the patch ECG monitors was 263.8 (SD 15.6) hours and of them, 84.3% of ECG signals were valid for appropriate interpretation, with noise or missing signals disregarded for accurate diagnosis of arrhythmic events (Table 5).

**Table 5:** Comparison of AT-Patch-recorded data between participants with and without atrial fibrillation (N=320).

ECG^a^		Value, mean (SD)	Atrial fibrillation by AT-Patch				Atrial fibrillation by AT-Patch or 12-lead ECG		
			Yes (n=11), mean (SD)	No (n=309), mean (SD)	*P* value	Yes (n=14), mean (SD)		No (n=306), mean (SD)	*P* value
Total recording time (hours)		263.8 (15.56)	240.2 (59.63)	264.6 (10.71)	.04	245.8 (53.47)		264.6 (10.76)	.10
Total valid time with noise (hours)		241.0 (45.33)	228.2 (70.41)	241.4 (44.28)	.50	234.7 (63.3)		241.3 (44.46)	.98
Total valid time without noise (hours)		206.7 (59.96)	188.5 (69.28)	207.4 (59.62)	.21	195.75 (63.8)		207.2 (59.84)	.31
Proportion of total valid time (%)		84.3 (17.13)	78.9 (20.55)	84.4 (17.01)	.28	80.4 (18.80)		84.4 (17.07)	.32
Proportion of noise time (%)		15.8 (17.13)	21.1 (20.55)	15.6 (17.01)	.28	19.6 (18.80)		15.6 (17.07)	.32
Normal sinus rhythm (%)		98.2 (4.22)	91.4 (8.99)	98.4 (3.75)	<.001	92.60 (8.27)		98.43 (3.77)	<.001
Ventricular ectopy (%)		0.5 (1.65)	5.2 (5.25)	1.2 (3.36)	<.001	2.84 (4.92)		0.37 (1.25)	<.001
Supraventricular ectopy (%)		1.3 (3.50)	3.4 (5.45)	0.4 (1.24)	.001	4.56 (4.83)		1.20 (3.37)	<.001
**Heart rate (beats/min)**									
	Mean	67.8 (8.60)	68.1 (10.38)	67.7 (8.55)	.98	67.4 (9.46)		67.8 (8.58)	.77
	Minimum	50.5 (7.51)	53.6 (9.26)	50.4 (7.44)	.24	52.6 (9.15)		50.4 (7.43)	.28
	Maximum	104.4 (17.51)	107.6 (29.78)	104.3 (16.98)	.75	105.9 (27.88)		104.3 (16.95)	.83

^a^ECG: electrocardiogram.

According to the patch analysis system, normal sinus rhythm was detected in 98.2% of patients, and the average heart rate of this study’s population was 67.8 (SD 8.6) per minute. The participants were divided into atrial fibrillation and the nonatrial fibrillation groups as classified by AT-Patch only and by AT-Patch or 12-lead ECG. When comparing the recorded ECG data of the atrial fibrillation and the nonatrial fibrillation groups classified by AT-Patch only and by AT-Patch or 12-lead ECG, both classifications similarly showed that supraventricular (3.4% vs 0.4%; *P*=.001 and 4.6% vs 1.2%; *P*<.001, respectively) and ventricular (5.2% vs 1.2%, *P*<.001 and 2.8% vs 0.4%; *P*<.001, respectively) ectopic rhythms were significantly more frequent in the new-onset atrial fibrillation group.

### Clinical Outcomes and Adverse Events

Few events occurred during the 6-month follow-up period, with no significant differences between the atrial fibrillation and nonatrial fibrillation groups (Table 6).

**Table 6:** Clinical outcomes and adverse events.

		Total, n (%)	Atrial fibrillation		
			Yes (n=11), n (%)	No (n=309), n (%)	*P* value
Stroke		2 (0.3)	1 (9.1)	1 (0.3)	.09
MI^a^		0 (0)	0 (0)	0 (0)	N/A^b^
Death		4 (1.3)	1 (9.1)	3 (1)	.32
Systemic embolism		0 (0)	0 (0)	0 (0)	N/A
**Adverse skin event**					
	Itching	13 (4.1)	0	13	>.99
	Rash	7 (2.2)	0	7	>.99

^a^MI: myocardial infarction.

^b^N/A: Not available.

**Figure 4:**
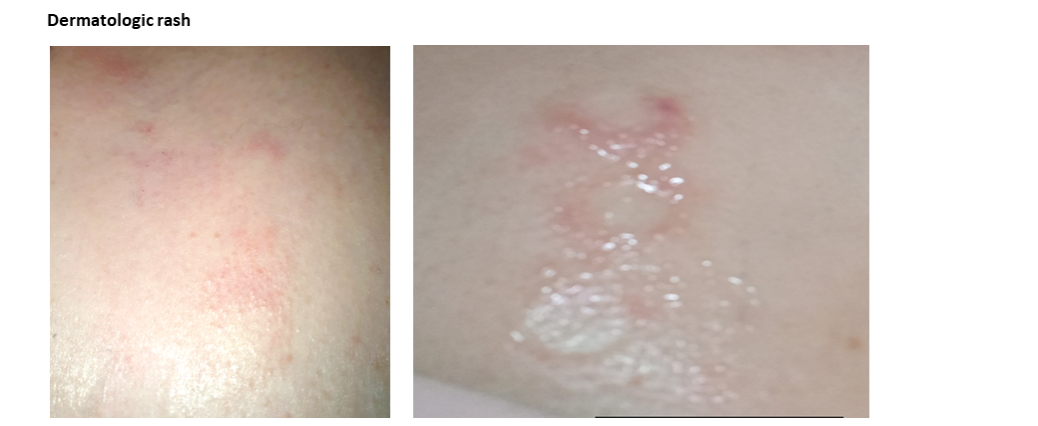
Adverse skin reactions due to the use of the AT-Patch.

Regarding adverse events affecting the skin, 4.1% (13/320) of participants were affected by itching during the patch monitoring period, and only 2.2% (7/320) of patients experienced a mild rash (Figure 4). All participants who experienced these adverse events recovered quickly without scarring or altered pigmentation.

## Discussion

### Principal Findings

This study aimed to demonstrate the diagnostic value of the AT-Patch, a wearable ECG monitoring device, for the detection of new-onset atrial fibrillation in high-risk patients. The results demonstrate the detection of a moderate number of new-onset atrial fibrillations among high-risk participants, with very few dermatologic reactions reported. These findings support previous studies that indicate that prolonged conventional ECG monitoring increases the detection rate of paroxysmal atrial fibrillations [16]. Further analyses of the new-onset atrial fibrillation group compared to the nonatrial fibrillation group demonstrated that a history of heart failure was the key feature among the CHA_2_DS_2_-VASc components that were highly correlated with atrial fibrillation. As the AT-Patch has been shown to be comparable to conventional devices in detecting arrhythmia, it is now essential to find predictors associated with high diagnostic identification of new-onset atrial fibrillation among high-risk patients [13]. With regards to this, almost half of the patients in this study with new-onset atrial fibrillation had a CHA_2_DS_2_-VASc score ≥4, including a history of heart failure. The pathophysiological relationship between heart failure and atrial fibrillation is not yet completely understood. However, many disease processes that predispose patients to heart failures, such as hypertension, diabetes mellitus, ischemic heart disease, and valvular heart disease, are risk factors for the development of atrial fibrillation [17]. Moreover, neurohormonal activation, cellular adjustment, and electrophysiological changes may create an environment that predisposes patients to the development of both heart failure and atrial fibrillation [18], with several studies reporting that the prevalence of atrial fibrillation increases as the severity of heart failure increases [19,20]. Therefore, the use of patch devices could be recommended for high-risk patients with a CHA_2_DS_2_-VASc score ≥4 and a history of heart failure.

The ECG data showed that the baseline electrical rhythm was more unstable in the atrial fibrillation group and that the occurrence of supraventricular and ventricular ectopic signals was more frequent in the new-onset atrial fibrillation group than in the nonatrial fibrillation group. Previous studies have indicated that episodes of atrial tachycardia potentially promote the remodeling of pulmonary vein cardiomyocytes and the left atrium, which may in turn be related to the arrhythmogenesis of paroxysmal atrial fibrillation [21]. Although atrial ectopic episodes appear to be more closely related to arrhythmogenesis in atrial fibrillation, the presence of premature ventricular complexes has been reported to be a significant predictor of paroxysmal atrial fibrillation [22]. This suggests that supraventricular and ventricular ectopic rhythms correspond to adverse changes throughout the atria and ventricular myocardium and can cause predisposition to the development of atrial fibrillation. The AT-Patch is small and causes minimal interference with the patient’s daily activities. As such it may be useful to repeat patch ECG monitoring in patients with frequent anomalous beats who present for the early detection of atrial fibrillation.

It is thought that the increasing incidence and prevalence of atrial fibrillation is in part due to the rapidly growing number of older adults in Korea [23]. A large proportion of unexplained strokes potentially originate from paroxysmal atrial fibrillation [24] and in addition, the burden of atrial fibrillation is associated with an increased risk of ischemic strokes [25]. Therefore, the early detection of new-onset atrial fibrillation is crucial for the prevention of adverse outcomes. The AT-Patch proved to be effective in prolonged ECG monitoring and the detection of new-onset atrial fibrillation in high-risk patients. However, further studies are required to determine how effectively early detection of atrial fibrillation by this patch can reduce adverse clinical outcomes, including death, stroke, systemic embolic events, and myocardial infarction, by increasing the follow-up periods. Higher-risk patients whose CHA_2_DS_2_-VASc score is ≥4 and who have a history of heart failure are candidates that should be investigated in relation to atrial fibrillation.

### Study Limitations

Despite our study being statistically designed to demonstrate a higher diagnostic yield of new-onset atrial fibrillation, it is possible that the sample size may not be sufficient to prove the diagnostic value of this wearable device. To overcome this limitation, we employed the Firth logistic regression for rare variant association tests to generate a more accurate and reliable estimate of the predictor variables (Table S2 in Multimedia Appendix 1). Our results using the Firth method indicate that heart failure is a reliable predictor for the diagnosis of new-onset atrial fibrillation, which supports the main argument of our study. As such, the selection of higher-risk participants may have prevented the recruitment of a sufficiently large sample size. Future studies of the atrial fibrillation group should have an increased focus on participants with additional risk factors, including a history of heart failure.

This study was designed to be completed after the follow-up period at 6 months and as such, we were unable to investigate the late clinical outcomes for the participants. Some studies have reported a substantially increased risk of stroke in patients discharged from the hospital with a diagnosis of atrial fibrillation in the first year after diagnosis [26]. However, as yet there are no reports regarding high-risk patients with new-onset atrial fibrillation detected during daily life. In addition, it is unclear how beneficial the use of anticoagulation therapy is in reducing adverse outcomes. As such, we are interested to further investigate long-term follow-up for any adverse outcomes following the initial diagnosis of atrial fibrillation.

### Conclusions

This study successfully detected a moderate number of new-onset atrial fibrillations using the AT-Patch device. This noninvasive, continuous ambulatory ECG monitor, which is less cumbersome to wear than conventional ECG monitors, is potentially beneficial for the early detection of atrial fibrillation and the prevention of subsequent adverse outcomes. Further studies will aim to investigate the relationship between heart failure and atrial fibrillation and use longer follow-up periods to determine how effectively prolonged monitoring with this patch device reduces adverse clinical outcomes of atrial fibrillation.

## Appendix

Multimedia Appendix 1 Supplementary table.

## Abbreviations

CHA_2_DS_2_-VASc: congestive heart failure, hypertension, age ≥75 years, diabetes mellitus, stroke, vascular disease, age 65-74 years, sex scale
ECG: electrocardiogram
