# Supplementary material for: Diagnostic Value of a Wearable Continuous Electrocardiogram Monitoring Device (AT-Patch) for New-Onset Atrial Fibrillation in High-Risk Patients: Prospective Cohort Study
Source: J Med Internet Res. 2023 Sep 18;25:e45760. doi: 10.2196/45760 (PMC10546264; doi:10.2196/45760)
Supplement: Multimedia Appendix 1 [file jmir_v25i1e45760_app1.docx]

**Supplementary Table**

**Table S1.** Each event of atrial fibrillation detected by AT-Patch.

| Subject No | AF starting time | AF termination time | Average heart rate (beat/min) during AF | Average heart rate (beat/min) during sinus rhythm | Longest AF event time |
| --- | --- | --- | --- | --- | --- |
| 1 | 2021-02-05 14:05:00 | 2021-02-05 18:50:00 | 64.77 | 63.03 | 2hr 45min |
|  | 2021-02-06 1:45:00 | 2021-02-06 1:51:00 | 56.09 |  |  |
|  | 2021-02-06 3:21:00 | 2021-02-06 3:55:00 | 47.38 |  |  |
|  | 2021-02-06 8:53:00 | 2021-02-06 8:56:00 | 61.25 |  |  |
|  | 2021-02-10 22:25:00 | 2021-02-10 22:53:00 | 60.85 |  |  |
| 2 | 2021-03-04 11:47:01 | 2021-03-04 15:18:21 | 73.76 | 60.75 | 3hr 31min 20sec |
|  | 2021-03-05 10:16:10 | 2021-03-05 11:16:00 | 73.87 |  |  |
|  | 2021-03-05 11:16:08 | 2021-03-05 11:21:30 | 86.22 |  |  |
|  | 2021-03-10 9:48:12 | 2021-03-10 10:17:00 | 72.28 |  |  |
| 3 | 2021-04-14 16:08:38 | 2021-04-15 2:40:35 | 83.98 | 63.86 | 15hr 50min 36sec |
|  | 2021-04-22 14:41:43 | 2021-04-23 6:32:19 | 85.93 |  |  |
| 4 | 2021-08-02 7:48:50 | 2021-08-02 7:50:41 | 132.14 | 75.66 | 1hr 10min 24sec |
|  | 2021-08-03 5:30:43 | 2021-08-03 9:17:07 | 129.54 |  |  |
|  | 2021-08-04 18:21:29 | 2021-08-04 20:01:17 | 105.44 |  |  |
|  | 2021-08-06 5:47:13 | 2021-08-06 6:58:28 | 109.89 |  |  |
|  | 2021-08-08 11:55:28 | 2021-08-08 13:53:28 | 102.14 |  |  |
| 5 | 2021-10-15 3:24:00 | 2021-10-15 5:44:50 | 74.18 | 84.09 | 2hr 20min 50sec |
| 6 | 2021-12-22 0:31:51 | 2021-12-22 12:18:10 | 94.72 | 92.86 | 11hr 36min 19sec |
| 7 | 2022-04-02 17:56:10 | 2022-04-02 20:43:00 | 69.27 | 62.91 | 2hr 46min 50sec |
|  | 2022-04-03 22:43:50 | 2022-04-03 22:55:07 | 71.56 |  |  |
| 8 | 2021-06-12 7:20:09 | 2021-06-12 7:44:37 | 100.14 | 74.96 | 24min 28sec |
| 9 | 2021-07-02 3:56:30 | 2021-07-02 4:01:32 | 73.48 | 87.03 | 5min 02sec |
| 10 | 2021-07-20 21:52:28 | 2021-07-21 1:23:24 | 74.67 | 73.99 | 3hr 30min 56sec |
| 11 | 2021-11-19 8:03:11 | 2021-11-19 18:36:02 | 80.99 | 82.05 | 10hr 32min 51sec |

**Table S2.** Results from Firth logistic regression for rare variant association tests.

| **Variables** | **Odds Ratio** | | **95% CI** | ***P-*value** |
| --- | --- | --- | --- | --- |
| Age | 0.94 | | (0.83-1.07) | 0.350 |
| Sex | 0.68 | | (0.07-5.21) | 0.719 |
| BMI (kg/m^2^)  Heart rate (beat/min) | 1.12  0.97 | | (0.92-1.38)  (0.87-1.06) | 0.227  0.474 |
| Hypertension | | 1.51 | (0.28-11.93) | 0.655 |
| Diabetes mellitus | | 1.89 | (0.37-11.33) | 0.447 |
| Dyslipidemia | | 0.71 | (0.15-3.24) | 0.646 |
| Previous MI | | 0.4 | (0.02-3.45) | 0.419 |
| Heart failure | | 5.41 | (0.82-46.04) | 0.078 |
| History of stroke | | 0.87 | (0.03-8.85) | 0.916 |
| CKD | | 2.62 | (0.17-18.44) | 0.415 |
| Current smoker | | 0.53 | (0.04-13.31) | 0.646 |
| Non smoker | | 1.58 | (0.14-43.54) | 0.732 |
| CHA2DS2-VASc≥3 | | 0.27 | (0.01-2.72) | 0.278 |
| Medications  Aspirin | | 0.66 | (0.12-3.41) | 0.618 |
| P2Y12 inhibitors | | 2.4 | (0.45-14.38) | 0.297 |
| ACE inhibitor  ARB | | 0.67  2.9 | (0.09-4.1)  (0.51-2.16) | 0.673  0.228 |
| Beta-blocker | | 0.49 | (0.09-2.16) | 0.342 |
| Calcium channel blocker | | 0.4 | (0.04-2.1) | 0.297 |
| Diuretics | | 1.01 | (0.11-6.46) | 0.989 |
| Statin | | 4.29 | (0.42-172.73) | 0.247 |
| Ventricular ectopy | | 1.37 | (1.08-1.83) | 0.009 |
| Supraventricular ectopy | | 1.16 | (1.02-1.34) | 0.022 |

**Backward elimination.**

|  | OR | 95% CI | *P-*value |
| --- | --- | --- | --- |
| BMI (kg/m^2^) | 1.2 | (1.02-1.38) | 0.028 |
| Heart failure | 7.8 | (1.78-30.96) | 0.008 |
| ACE inhibitor | 4.44 | (1.02-17.78) | 0.048 |
| Ventricular ectopy | 1.37 | (1.15-1.67) | 0.001 |
